# Learning to Decompose: Human-Like Subgoal Preferences Emerge in Neural Networks Learning Graph Traversal

**DOI:** 10.1162/OPMI.a.256

**Published:** 2025-11-10

**Authors:** Yuxuan Li, James L. McClelland

**Affiliations:** Department of Psychology, Stanford University, Stanford, CA, USA

**Keywords:** task decomposition, subgoal, neural network, learning, cognition

## Abstract

Cognitive scientists have discovered normative and heuristic principles that capture human subgoal preferences when partitioning problems into smaller ones. However, it remains unclear where such preferences come from and why they tend to be both effective and efficient. In this work, we study the processes through which these preferences may be implicitly encoded over learning as learners improve towards optimal traversals. We build on the graph-based environments from prior work and use neural networks as model learners to test if learning shortest-path traversal can lead to human-like path decomposition. We find that simple transformer models develop a preference for paths containing nodes that occur frequently on the shortest paths, consistent with human subgoal preferences found in prior work. This preference is observed when models solve shortest path traversals for unseen problems in both known graphs and new graphs, demonstrating that human-like subgoal preferences can arise without requiring explicit preference computation or exhaustively searching over all possible paths. The same preference does not emerge when models learn to perform random or Hamiltonian traversals. Our findings are robust across several transformer variants as well as recurrent neural networks, suggesting they depend more on the data distribution than the network architecture.

## INTRODUCTION

Humans can often break larger tasks and goals into smaller ones, for example, cooking a meal through multiple steps, organizing a day’s work into different chunks and themes, or planning a long trip via smaller segments. Such efficient ways of achieving higher-level goals have long been a core topic of interest in cognitive science (Botvinick et al., 2009; Miller et al., 1960). Researchers have designed controlled experiments to study human task decomposition and subgoal choices, finding that humans exploit task structures to simplify tasks, construct multi-level action plans, and show consistent subgoal preferences (Correa et al., 2023; Eckstein & Collins, 2020, 2021; Huys et al., 2015; Solway et al., 2014; Tomov et al., 2020). These behavioral signatures are complemented by neural representations that reveal how the brain tracks subtask contexts and discovers environment structures (Balaguer et al., 2016; Schapiro et al., 2013).

In humans, the tendency to decompose tasks and select good subgoals reflects computational advantages. Recent work showed that human subgoal preferences in graphs of connected states in fact satisfy constraints that can be mathematically characterized (Correa et al., 2023; Solway et al., 2014; Tomov et al., 2020). In these experiments, participants typically undergo a learning phase to familiarize themselves with a graph-structured environment, for example, through learning edges (e.g., identify neighbors of a given node) or through navigation tasks (e.g., perform traversal given a start and goal pair). After the learning phase, participants are evaluated on their subgoal choices in a navigation setting from pairs of start and goal nodes. Behavioral evidence suggested that human participants tend to consistently identify certain states in the graphs as subgoal choices. In a recent experiment, Correa et al. (2023) collected such choices from diverse graphs and compared them with prior accounts, finding that human choices were most consistent with a graph property called Betweenness Centrality. That is, people’s subgoal choices favor states that frequently appear on the shortest paths in the graph.

Empirical human subgoal choices in these graph-like environments turned out to nicely align with normative principles of choice optimality and efficiency. For example, people tend to identify bottleneck nodes in graphs as subgoals or derive graph decompositions that optimally represent shortest path trajectories (Solway et al., 2014). Tomov et al. (2020) showed that human state partitions not only reflect the topology of the environment, but also cluster together states that frequently co-occur or states that have similar reward profiles. Correa et al. (2023) further pointed out that human subgoal choices accord with a resource-rational consideration, reducing the cost of action planning when determining how the graph environment is decomposed.

Although these normative principles closely capture human subgoal choices as an end-product, it remains unclear what cognitive processes implement these choices or where these choice preferences come from. Importantly, explicitly tracking normative objectives such as the optimality of all possible decompositions or costs of different solutions requires expensive computation at inference time. As also noted in Correa et al. (2023), even if we assume that humans implement a strategy based on Betweenness Centrality to select subgoals, computing exact node Betweenness Centrality is still nontrivial and requires explicit, online knowledge of all shortest paths in a given graph.

**Figure 1.**
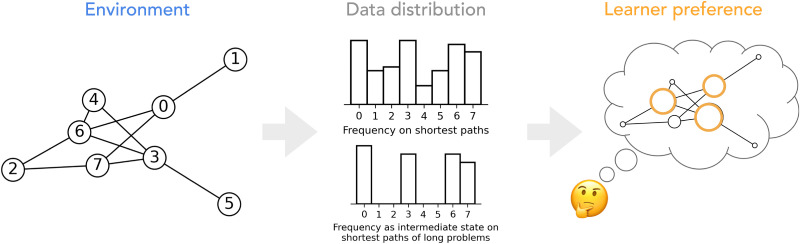
**The structure of the environment shapes the data distribution of optimal solutions, which may lead learners to develop emergent preferences.** (A) An example graph environment with eight states. (B) The structure of the environment governs the data distribution of good solutions, for example, the state frequency on efficient traversals, and candidates of optimal solutions to difficult tasks (e.g., long traversal). (C) When learning to approximate these optimal distributions, learners such as humans or models can acquire implicit preferences consistent with the properties of the environment.

In this work, we explore whether human-like subgoal preferences can arise from a more tractable computational process: as a result of learning to efficiently traverse graph-like environments. We were particularly motivated by the close alignment between human subgoal choices and Betweenness Centrality (BC) found in Correa et al. (2023). As illustrated in Figure 1, the inherent structure of the environment shapes the distribution of optimal solutions. Learners may thus develop preferences aligned with the environment properties as they integrate over experiences and optimize towards more efficient solutions (i.e., acquiring sensitivity to high BC nodes and coming to choose paths with such nodes over other paths).

We use neural networks as the model learning system to explore the interplay between environment structure, optimal solution distribution, and learned preferences. We ask whether models trained to efficiently traverse the graph will develop the same path and subgoal preferences seen in humans. Importantly, these models offer an appealing candidate of how human-like subgoal choices may be generated without exactly computing BC or other normative objectives. At inference time, node and path predictions are determined by selecting the alternative with the strongest activation through the forward propagation across the model layers. Any particular preferences that the model may acquire (such as a sensitivity to node BC values) are implicitly “cached” within the model weights throughout the course of learning. Viewed another way, the computation cost for choosing good subgoals does not solely incur at decision time, but is offloaded over the history of learning as the learner optimizes its behavior.

We find that, after training, neural network models successfully learn shortest path prediction in graph-structured environments. Moreover, they develop a preference for predicting paths that contain more high BC nodes over other equally short candidate paths, consistent with human subgoal choices observed in similar graph environments. We show that this preference is observed when models solve efficient traversal on new problems in graphs previously encountered and on new graphs not encountered during training. Since models were not exposed to any traversal experiences in those generalization settings, our findings indicate that they acquire implicit sensitivity to BC without exactly computing it over novel graphs encountered in context at inference time.

We also show that the human-like preference for BC is robust to superficial changes in data statistics, replicating prior evidence that human subgoal choices do not rely on simple state frequency over the learning history (Correa et al., 2023). In our experiments, we focus on simple transformers (Vaswani et al., 2017), as earlier work showed that transformers can acquire sensitivity to structures in the training data and even develop the ability to decompose tasks (Chan et al., 2022; Li & McClelland, 2023; Manning et al., 2020). We also explore transformer variants with different token prediction strategies or graph representations as well as recurrent neural networks, and contrast behavior of models trained to efficiently traverse with models trained on alternative forms of traversals. Taken together, our results suggest that a preference for BC can arise in a variety of model types and depends on the statistical properties of efficient paths.

We leverage the availability of the model learning profile to study the development of model path preferences over time, and discuss how the environment introduces different learning pressures that result in both rapid, effective learning and early biases. We suggest that similar learning dynamics may occur in human learning profiles, opening up new directions for future experimental work. Beyond model path preferences, we also explore ways to elicit more overt subgoal choices from some model variants to see how well such choices align with human subgoal choices. Finally, we discuss how this learning perspective can be extended to account for related behavioral phenomena, by considering factors that may alter the data-distributional properties of the optimal solutions.

## METHODS

### Dataset

We use two graph datasets containing 8-node and 15-node graphs to explore model behavior when learning efficient graph traversal. The 8-node graph dataset contains the 30 graphs used in the human experiment in Correa et al. (2023). We created the 15-node graph dataset to test if the same results will generalize to larger graphs and explore model behavior on longer paths. The 15-node graph dataset contains 50 simple, connected, undirected graphs. These graphs are randomly generated using the Erdős-Rényi model with an edge probability of 0.25, and constrained to have an edge-to-node ratio between 1.1 and 1.6 using rejection sampling.

**Figure 2.**
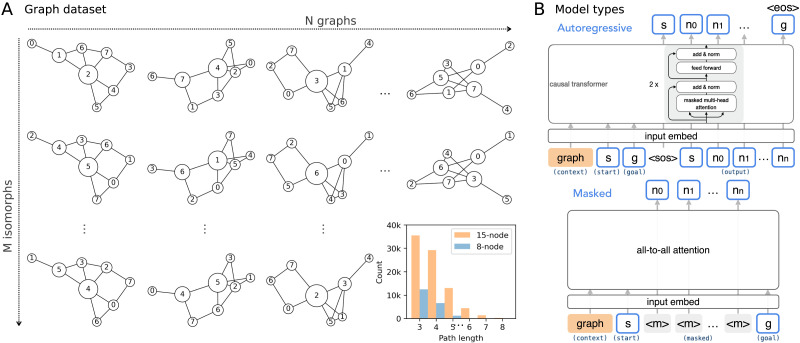
**Shortest path traversal task and model architecture.** (A) Example graphs in the 8-node graph dataset. Node sizes are scaled to reflect the node Betweenness Centrality score. The lower right panel shows the distribution of shortest path lengths for all unique start-goal pairs in both datasets. (B) Top, the autoregressive transformer predicts the shortest paths one node at a time from the start node to the goal node. Bottom, the masked transformer predicts the masked intermediate nodes on the shortest paths in one forward pass during training, and generates all intermediate nodes one at a time by predicting the node with the maximum negative output entropy during evaluation.

We generated 10 isomorphic instances for each unique graph, thus totaling 300 and 500 different graphs in each graph set (Figure 2A). The dataset includes all unique start-goal pairs in these graphs with at least one intermediate node (see the lower right panel in Figure 2A for the path length distribution of the shortest paths associated with these start-goal pairs).

### Model

The models are trained to predict nodes on a shortest path that connects a start node to a goal node within a given graph (Figure 2B, see more on path sampling below). We use a graph token in the form of a set of learnable graph embeddings to indicate graph information, where each embedding represents one isomorphic instance of a unique graph connectivity. The node labels are expressed through node tokens corresponding to a set of learnable node embeddings (shared across all graphs). When appearing as start or goal nodes, a learnable start embedding and a learnable goal embedding are added to the node embedding.

We first train a standard decoder-only transformer to generate nodes on the shortest path autoregressively (Figure 2B, top). These models use all-to-all attention between the graph token, start node, and goal node, then begin the autoregressive, future-masked prediction with a fourth start-of-sequence token with an all-zero embedding. Starting with the start node token, the prediction is followed by the intermediate nodes on the shortest path, and finishes by generating an end-of-sequence prediction simultaneously with predicting the goal node token. The autoregressive models are trained using teacher-forcing and evaluated with top1 rollout (greedy decoding). We use autoregressive transformers with two layers of standard attention blocks each containing single-headed self-attention and feed-forward sublayers, using an embedding dimension as well as a hidden dimension in the feed-forward sublayer of 256. We use this architecture as it was the minimally effective architecture that achieved high prediction accuracy on held-out paths compared to a range of other architectural hyperparameters (see Figure S2 in the Appendix for additional details).

Because the autoregressive models always predict the shortest path in a restricted start-to-goal order, they do not permit studying explicit model subgoal choices during path generation. We therefore also investigated a masked transformer that supports a more dynamic path prediction process (Figure 2B, bottom). The masked models are trained using masked path completion during training, where attention between all tokens in the context is allowed. Each time a shortest path with *n* intermediate nodes appears in a training batch, we uniformly sample 1 to *n* intermediate nodes at random positions to mask out. The masked models are trained to predict the masked-out nodes in a single forward pass using the graph token, start and goal nodes, and the non-masked intermediate nodes as context. During evaluation, we mask out all intermediate nodes and apply an iterative path completion procedure to generate nodes on the output path. For a path containing *n* intermediate nodes, the models undergo *n* forward passes, each time predicting the node with the maximum negative entropy (Mansimov et al., 2019). All node tokens, including the start node, goal node, and intermediate nodes (masked or unmasked), are added with learnable position embeddings to supply token distance information relative to the start node and the goal node. The masked models have only one layer (which, at inference time, is repeatedly applied to generate the intermediate nodes on the path) and otherwise have the same architecture as the autoregressive transformers.

In addition to autoregressive and masked transformers trained on graph tokens, we also replicate the main findings on two additional model families: 1) a transformer variant that generalizes to novel graphs with edge token inputs, and 2) recurrent neural network models, specifically the Long Short-Term Memory network (LSTM) (Hochreiter & Schmidhuber, 1997). The edge-token transformers have the same architecture as the graph-token transformers, but receive more input tokens. For the LSTM, we use a one-layer model and use 512 for both the embedding dimension and the hidden dimension to increase its performance. Both the autoregressive edge-token transformer and the LSTMs are trained using teacher-forcing and evaluated using rollout.

### Training and Evaluation

We train all model families using a random 75% of all start-goal pairs in each dataset during training, and hold out the remaining start-goal pairs for evaluation. Importantly, each time a start-goal pair with multiple shortest paths appears during training, we uniformly sample a candidate path to supervise the model to ensure equal exposure to all valid answers. We use a batch size of 128 to train the models and use the Adam optimizer for gradient update with a learning rate of 0.0003. Training lasted for ∼30k gradient steps on the 8-node graph dataset and ∼200k gradient steps on the 15-node graph dataset. For all results shown below, we evaluate models on the held-out start-goal pairs whose shortest paths contain at least two intermediate nodes, similar to prior work (Correa et al., 2023). All results are aggregated across four model seeds. Unless otherwise noted, all error bars indicate the standard deviation over model seeds.

### Open Source

The modeling and analysis code used in this paper is available in this GitHub repository: https://github.com/Effie-Li/graph-subgoal-public.

## RESULTS

**Figure 3.**
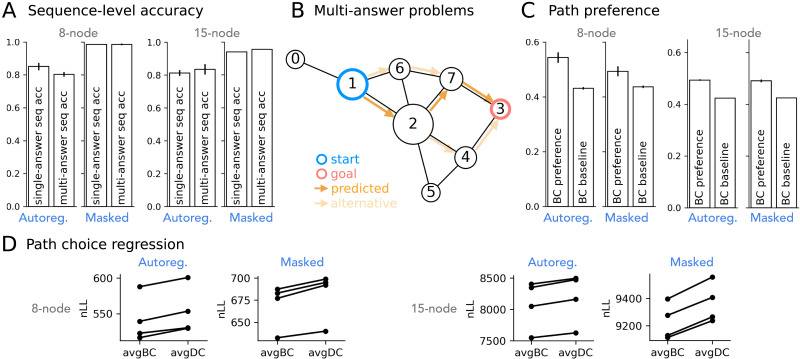
**Model-predicted shortest paths show human-aligned preference.** (A) Sequence-level accuracy of predicted shortest paths for unseen start-goal pairs (with at least two intermediate nodes). (B) An example multi-answer problem with three equally short paths. (C) For multi-answer problems, model-predicted shortest paths show preference for Betweenness Centrality (BC). BC preference: the proportion of model-predicted shortest paths that have the highest average node BC compared to alternative shortest paths. BC baseline: the preference score under randomly chosen shortest paths. (D) Betweenness Centrality better predicts model path selection over candidate shortest paths. Each line shows one model seed. avgBC: average Betweenness Centrality for intermediate nodes on a shortest path. avgDC: average Degree Centrality.

We studied whether neural network models trained to predict shortest paths in graph-like environments learn human-like preferences in their predictions. Transformer models successfully learned to predict shortest paths in both the 8-node and 15-node graph datasets (Figure 3A). Autoregressive models generalized to held-out start-goal pairs at 80% sequence-level accuracy (1 = correctly predicted all intermediate nodes, 0 = otherwise). Masked models achieved even higher sequence-level accuracy at 98% using iterative path completion.

The key behavior of interest is whether the models developed a consistent path preference in multi-answer problems, i.e., start-goal pairs with multiple shortest paths (Figure 3B). Previous behavioral experiments showed that humans tend to identify states with high Betweenness Centrality (BC) as subgoals in graph-like environments (Correa et al., 2023). If the models learned similar preferences, we should expect that they consistently predict paths containing more high BC nodes over other candidate paths for multi-answer problems. We note again that all candidate shortest paths associated with each multi-answer problem are uniformly sampled to supervise the model during training.

### Model Path Preferences

Models indeed showed preferences for paths containing more high BC nodes. Figure 3C shows that the model-predicted shortest paths more often have the highest average BC across intermediate nodes (BC preference, conditioned on correct predictions only) compared to randomly chosen shortest paths (BC baseline). We used a path regression method similar to Correa et al. (2023) to test how the average BC of intermediate nodes on a path quantitatively tracks model path choice compared to an alternative centrality property, Degree Centrality (DC), using log average BC or DC as the path predictor. This analysis confirmed that BC is a better predictor for both the autoregressive and the masked models’ path choices (Figure 3D). Although the effects are small (in part due to the nature of the graphs such that paths with high average BC and paths with high average DC often coincide), they are consistent across seeds and appear statistically reliable (Bayes Factors for regression pairs across all seeds > 1717).

### Centrality Preference Over Learning

**Figure 4.**
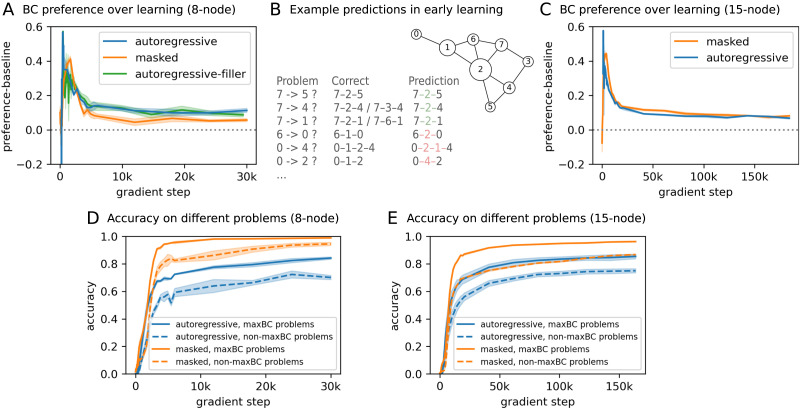
**The development of model path preference over learning.** (A) Betweenness Centrality (BC) preference over learning for models trained on 8-node graphs. BC preference (baseline adjusted) measures how often the correct shortest path predicted by the model has the highest average node BC among shortest path candidates, compared to a randomly chosen shortest path. Error shades indicate the standard error of the mean. Autoregressive-filler models are trained with the modified dataset containing filler paths to balance node visitation (see main text). (B) Model predictions for example held-out start-goal pairs in one 8-node graph in early learning where BC preference peaks. (C) BC preference (baseline adjusted) over learning for models trained on 15-node graphs. (D–E) Accuracy on held-out problems with at least two-intermediate nodes over learning, for start-goal pairs with a graph max BC node in any of its shortest paths (maxBC problems) and those without (non-maxBC problems).

We showed that these models develop a preference for predicting shortest paths containing high BC nodes after training. A natural next question is: how did this path preference develop as models learned the task over training? We evaluated the BC preference and BC baseline metrics over checkpoints throughout model learning. Both autoregressive and masked models showed a strong surge in their BC preference early in learning, followed by a gradual decrease, before settling to a lower final preference level that is still noticeably above baseline (Figures 4A and 4C).

High BC nodes, by definition, are most frequently encountered across all shortest path problems in a given graph. This means that even though we ensured equal exposure to candidate shortest paths for any given problem during model training, models can learn a global, graph-level bias for high BC nodes by integrating shortest paths over multiple problems. This bias can strongly influence prediction on individual start-goal pairs, leading the model to learn paths through these nodes first. In fact, models not only learn such path preference for a given problem, but learn to predict start-goal pairs with a graph max BC node in any of the shortest paths earlier and more accurately than other problems (Figures 4D and 4E, note additionally that start-goal pairs with a graph max BC node in the solutions can be more prevalent than other problems among long traversal problems, up-weighing the overall accuracy). However, the same bias can also lead to errors in wrongly predicting high BC nodes on irrelevant paths (Figure 4B). Over time, this preference is balanced with learning the correct predictions for more start-goal pairs.

### Betweenness Centrality vs. Simple Node Frequency

Node Betweenness Centrality necessitates entanglement with node frequency in a shortest path distribution. The high sequence-level accuracy on held-out problems (i.e., predicting all intermediate nodes correctly and in the right order) suggests that models are not simply predicting the most frequently-encountered node on any path for a given graph. Prior human experiments showed that human subgoal preferences are robust to simple node occurrence frequency (Correa et al., 2023). Here, we use a control experiment to further show that model path preferences are similarly robust to superficial node frequency over the learning history.

We disentangle simple node occurrence frequency from node BC during model training by augmenting the shortest path dataset with a set of filler problems (see Appendix A for additional details on generating filler problems). These filler paths serve to balance node frequency throughout training, similar to the length-2 filler problems (i.e., an edge in the graph) used in the human experiments in Correa et al. (2023). As shown in Figure 4A, the path preference of models trained on this augmented dataset replicated the results from models trained on the original dataset. This shows that models’ sensitivity to node BC is robust to superficial node frequency over learning, echoing the human preferences mentioned above. We note that this analysis was only appropriate with autoregressive models. This is because the learning objectives of masked models concern only the intermediate nodes, leaving masked models unable to learn anything with the length-2 paths. We further reflect on the reason behind the robustness of this preference in the Discussion section below.

### Model Subgoal Choices

As alluded to earlier, the masked models iteratively generate the nodes on the output path one at a time by predicting the node of maximum negative entropy. This dynamic prediction process provides an opportunity to study masked models’ subgoal choices. We use the 15-node dataset to study this, as longer and more paths allow for more detailed analysis.

**Figure 5.**
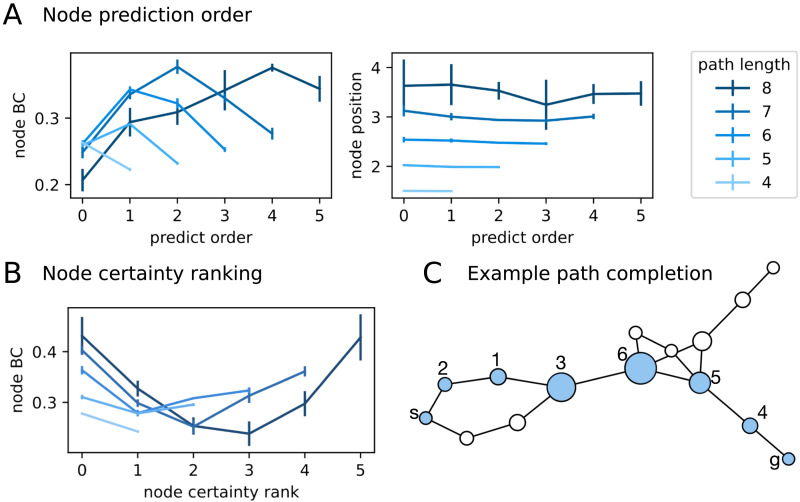
**Investigating masked models’ subgoal choices.** (A) Node prediction order, aggregated over held-out sequences of different lengths. Left, node Betweenness Centrality (BC). Right, node position on the output path. (B) Node certainty ranking. Node certainty is defined by the sum of node output probability across all intermediate positions. Colors indicate different path lengths (shares legend with panel A). (C) Example shortest path completion of an unseen length-8 problem that the model correctly predicted. Node sizes are scaled to reflect the node Betweenness Centrality score. s: start node. g: goal node. The numbers indicate the prediction order.

We first asked if masked models predict some nodes consistently earlier than others. As shown in Figure 5A, masked models’ node prediction order shows no preference for node position on the path, but shows consistent sensitivity to node BC. In particular, masked models tend to predict low BC nodes in either the beginning or the end, and predict high BC nodes in the middle of completing the output path. This pattern is observed across multiple path lengths in the held-out evaluation paths.

If we regard the first predicted node as the model’s subgoal choice for the path, then masked models’ subgoal choices did not align with human subgoal preferences. One possibility is that, even though masked models may have high certainty that a node should appear on the resulting path, this certainty is diluted over multiple positions, and concentrates after a few low BC nodes are filled into the path, especially those that bottleneck the start or the goal nodes. We thus created a certainty metric using summed node probability in the output distribution across all intermediate positions in the first forward pass during evaluation, i.e.,

$$\text{Certainty}\left(\text{node}\right)=\sum_{\begin{array}{c} \text{pos}\;\in \\ \text{interm}.\;\text{positions} \end{array}}P\left(\text{node}|\text{pos}\right)$$

When the nodes are sorted according to this certainty measure, we found that masked models were indeed more certain for the high BC nodes to appear on the output paths (Figure 5B). We note that the certainty metric is an empirical measure derived from model output predictions, although it could approximate the true marginalized probability that a node appears on any shortest path in that particular graph as a consequence of learning.

We further found that the iterative, maximum negative entropy-based procedure led to a nontrivial path completion process that combines local subpath completion with global path segment linking; one such example is shown in Figure 5C. This may explain why some high BC nodes are ranked low on the certainty measure, as they were predicted last to connect local path segments. Across the evaluation paths, models trained on the 15-node dataset predicted non-adjacent nodes for only ∼10% of sequentially predicted node pairs (where the first-predicted node is evaluated against the start or goal node). For shorter paths (e.g., paths containing two intermediate nodes), we observed that sequential prediction typically shows up as predicting the entire path in either the forward (start-to-goal) or backward (goal-to-start) direction. But the models show no overall bias in directionality, as the start, goal, and path directions are represented symmetrically in the masked models.

**Figure 6.**
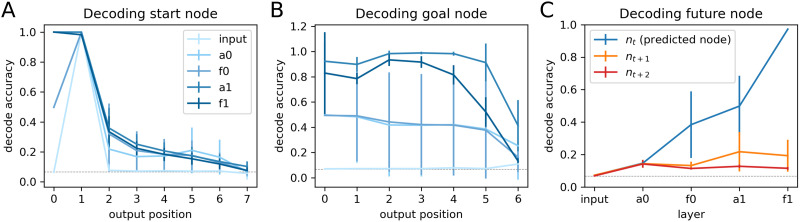
**Autoregressive models keep track of goal node information in intermediate layers.** (A–C) Decoding the start node, goal node, and future nodes from token representations across layers of the autoregressive models. In A and B, output position 0 corresponds to the start-of-sequence token as the input and start node as the target output. Input: input embedding. a0/a1: token states after the self-attention sublayer in each attention block. f0/f1: token states after the feed-forward sublayer in each attention block. The gray dashed lines indicate chance level (1 of 15 nodes).

We also explored to what extent autoregressive models are implicitly encoding the start node, goal node, or even future nodes to aid next node prediction. To examine this, we trained multi-class logistic regression classifiers to decode the start node, goal node, and future node identities on the paths from token representations across intermediate layers of the network (4-fold cross-validation, no regularization applied). As shown in Figure 6, autoregressive models quickly “forget” start state information as they generate nodes on the output path, but tend to represent goal information throughout path generation. For future nodes immediately following or two nodes after the to-be-predicted node, the models encoded limited information. We note that for decoding the goal node, we excluded nodes whose next node is the goal node. Similarly, for decoding future nodes, we excluded nodes whose future nodes coincide with the goal node.

### Other Architectures

**Figure 7.**
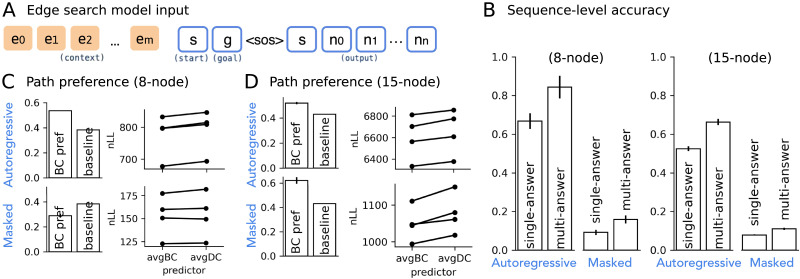
**Modifying model input to support generalizing shortest path prediction to unseen graphs.** (A) In edge search models, the graph context is supplied through a set of edge tokens (sum of two node embeddings) instead of a single graph token. (B) Sequence-level accuracy on start-goal pairs in unseen graphs for both model types on both graph datasets. (C–D) Path preferences of these edge search models.

The models discussed so far capture the essential learning behavior that can explain path and subgoal preferences in graph-like environments. However, they are limited in generalization: they only generalize to unseen start-goal pairs in known graphs but cannot predict shortest paths in novel graphs. We experimented with replacing the single graph token with a set of edge tokens to signal graph connectivity information to the model, where each edge token is the summed embeddings of two nodes (Figure 7A). This allows the model to perform in-context graph traversal, rather than learning to retrieve connectivity information implicitly through the graph embedding (see also Figure S3B for an example attention pattern showing the model’s learned in-context graph traversal). The autoregressive models with edge token input successfully predicted shortest paths for over 60% of the long problems (i.e., those with at least two intermediate nodes) in completely unseen graphs (Figure 7B). In addition, autoregressive edge search models replicated the preference to predict paths containing high BC nodes (Figures 7C and 7D). The masked edge search models, however, only correctly predicted less than 20% of the long problems in unseen graphs (Figure 7B). Despite the low accuracy, masked edge search models showed similar path BC preference for the novel paths they correctly predicted in the 15-node dataset. We suspect that these models will strongly benefit from increased model capacity, as deeper layers or parallel attention heads may support more effective in-context search over the edge tokens to achieve higher accuracy.

**Figure 8.**
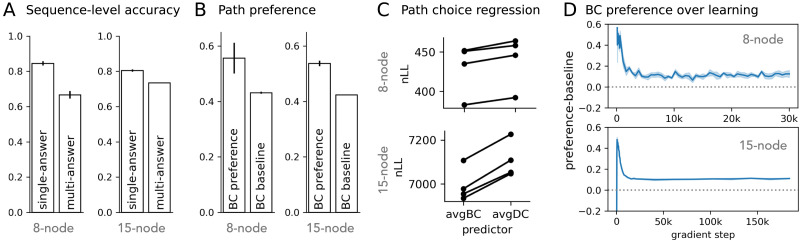
**LSTMs also learn similar human-aligned path preference.** (A–D) Sequence-level accuracy, path preferences, and preference over learning derived from LSTMs’ prediction of shortest paths for unseen start-goal pairs.

In addition, we show that the main findings on model-learned path preferences replicate in non-transformer architectures, for example, using LSTMs (Figure 8). The performance of the LSTMs is lower than the transformer counterpart using the same graph token input, even with more trainable parameters (from increased latent embedding dimension). However, for the correctly-predicted held-out problems, we observe similar learned preferences in LSTMs. In general, as we discuss below, these results suggest that any learning system with a reasonable capacity to learn an efficient traversal objective will likely acquire a non-trivial BC-aligned preference, as the environment properties directly influence the data distributions the systems may optimize towards.

### Alternative Learning Objectives

Lastly, we also explored whether similar preferences for Betweenness Centrality develop in models trained on alternative learning objectives and traversal tasks. We created alternative datasets for the 8-node and 15-node graphs that contain random walks that connect each start-goal pair. We also created a Hamiltonian walk dataset that contains Hamiltonian paths (paths that visit every node in the graph exactly once), using the 15-node graph in Schapiro et al. (2013). We then applied the same training across three model types (autoregressive transformers, masked transformers, and LSTMs) to study whether models trained under alternative traversal objectives (random or Hamiltonian) may exhibit some form of BC preference. The full data generation and experimental details are presented in Appendix D.

Overall, we do not find that models trained under these alternative learning objectives develop BC preferences (see quantitative results in Tables 1 and 2 in the Appendix). Models trained on random walks successfully learn to predict valid walks and sometimes predict a shortest path linking a start-goal pair. However, the shortest paths they predict do not favor paths with high average BC over other candidate shortest paths. Models trained on Hamiltonian walks also predict Hamiltonian walks reasonably well, but do not exhibit any bias for nodes with high BC in the errors they make. These results suggest that learning to familiarize with the graph connectivity through traversal experiences alone is not sufficient for human-like preferences to emerge. An explicit learning pressure to optimize for efficient traversal across many problems may be a key ingredient for such preferences to develop in a learning system.

## DISCUSSION

What might explain the origin of human subgoal choice preferences? Prior work in cognitive science has found that human subgoal choices in graph-like environments are closely related to Betweenness Centrality (BC), a graph property that measures how often a node appears on shortest paths (Correa et al., 2023). We explored whether human-like subgoal preferences can emerge in a learning system without explicit instructions to track BC or other normative objectives. Using neural networks as model learners, we examined the consequences of learning to perform efficient graph traversal. We show that, after training, the models successfully predicted shortest paths for unseen start-goal pairs and acquired a preference for predicting shortest paths containing high BC nodes, consistent with the preference observed in human subgoal responses.

Our modeling results suggest that learning and optimizing traversal solutions may serve as an implicit pressure for human-like subgoal preferences to arise. The models in our study are not trained to directly predict high BC nodes or compute node BC when generating the output. Rather, they acquire sensitivity to BC in their weights when optimizing the learning objective. While it is possible that some models may encode precise node BC information in their embeddings over training, we showed that models trained with in-context edge information generalize the same preference when efficiently traversing new graph environments. These results highlight that human-like, BC-aligned preferences can arise in a learning system without relying on precisely tracking the relevant graph properties or memorizing all shortest paths for a graph.

The model learning process offers a useful analog to compare and contrast how humans may discover good subgoal choices with more experiences in an environment. The data distribution of optimal traversals is constrained by the inherent structure of the environment. As shown in Figure 1, high BC nodes appear frequently among efficient traversals, especially on long traversal problems. Human learners may thus similarly acquire a sensitivity to BC as they improve and converge towards the optimal shortest path distributions. We also found that the model BC preference first increases in the early stages of learning and becomes less pronounced with extended training as models master more nuances and exceptions. These learning dynamics match a general feature of human learning in many domains (Rogers & McClelland, 2004; Rumelhart & McClelland, 1987; Seidenberg & McClelland, 1989). In future experimental work, it would be interesting to confirm whether similar temporal dynamics exist in human subgoal preferences over the course of learning.

Importantly, we show that models’ BC sensitivity is robust to superficial node frequency over the learning history, just like that observed in humans. One explanation of this robustness is that models learn most from the part of the training data distribution that contributes most meaningfully to the learning objective. Although the filler paths in the control experiment balanced the global node occurrence rate in the training data, they mostly consisted of length-2, single-edge filler problems. These single-edge filler problems may introduce weaker learning pressure compared to longer traversal problems, as the start and goal nodes are both presented in context. Such differences in learning pressure associated with different problems may similarly occur when humans learn from interleaved long traversal tasks and filler tasks. This would allow humans to disentangle simple node frequency from node frequency among candidate solutions important for efficient traversal when selecting good subgoal candidates.

There are a few important aspects of human learning that our modeling experiments did not directly capture. Below, we discuss how our general learning framework can be naturally extended to account for these different aspects.

First, for simplicity, models are directly supervised on shortest paths in our experiments. This allowed the models to quickly learn to efficiently traverse the graph environments, but we do not intend for this process to serve as an exact model of the human learning process. Humans learn navigation in a more interactive and exploration-based manner with different feedback (such as through reinforcement learning with rewards). We showed that, for models, simply learning to traverse the environment is not sufficient. Instead, experience traversing shortest paths is what drives the human-like preferences to emerge. Human learners may learn to sample shortest paths to minimize the discounting of rewards, leading them to develop sensitivity to useful intemediate states. This initial sensitivity may in return influence their experiences within the environment and reinforce these preferences as they eventually learn from more optimal data distributions. Investigating how subgoal preferences are formed within this kind of dynamic, bidirectional influence between the environmental and active exploration is an exciting topic to explore in future work.

We have also focused on cases where the optimal path distributions are directly tied to the topological centrality properties of the underlying graph. However, topology is not the only factor that may govern the optimal data distribution. For example, humans can develop subgoal preferences even in graphs with no centrality differences between different states (Tomov et al., 2020). Our learning account can be extended to explain how preferences develop in these settings, by considering the “effective” BC over the optimal solutions under different reward or task distributions. A non-uniform reward distribution can incentivize visiting some states (nodes) more frequently. A reward distribution aligned with topological BC may thus strengthen path and subgoal preferences based on topological BC, such that humans or models learn the preference earlier or show an even stronger preference. If the reward distribution does not align with topological BC (e.g., if the reward is high for a low topological-BC state), the reward will increase the apparent BC of states with low topological BC, thus weakening sensitivity to topological BC or competing with it during learning. Other extraneous factors such as task distributions can also shift the optimal path distribution (e.g., higher exposure to particular subgraphs). In such cases, considering the effective BC based on the particular task demand will be useful in understanding the learned preference.

Lastly, humans probably learn subgoal or path preferences at a much more data-efficient timescale, compared to our models that learn to represent graphs with graph tokens. Pure online learning during an experimental session may suffice for human participants to identify high BC nodes as good subgoal choices. That is, without any prior preference, participants may acquire a BC preference as they gain familiarity with the graph-like environment, as is common in the experimental paradigms used to study human subgoal choices (Correa et al., 2023; Solway et al., 2014; Tomov et al., 2020). This can potentially correspond to early steps in model learning in our study, where strong BC preference is observed before mastering optimal traversals within the target graphs. An interesting future direction would be to train models that learn to use a small number of traversals of a new graph in context, similar to the number of traversals participants in experiments see. Our results also predict that the BC-based subgoal preference would decrease as humans further gain familiarity with the environment and master optimal traversal. Outside of an experimental context, it is also possible that a BC-based preference truly manifests in physical or abstract traversal tasks we encounter in everyday life, such as navigating a city, or learning to reason with problems conceptually resembling states on a graph. We hope that our work motivates future experiments to investigate the learning dynamics of human subgoal choices in different task spaces.

On a more machine learning note, although transformers can be applied to general graph datasets and predict missing nodes or community properties (Dwivedi & Bresson, 2020; Ying et al., 2021), researchers have only recently begun to understand how transformers may perform graph reasoning *per se* (Brinkmann et al., 2024; Cohen et al., 2025; Jelassi et al., 2024; Sanford et al., 2024). Our work adds to these efforts that study transformer capabilities and internal computations with a data-distributional and human-alignment perspective. We also note that two factors were likely essential for the masked models to outperform autoregressive models on the shortest-path prediction task (on unseen start-goal pairs within known graphs). First, masked models receive privileged information on how many intermediate nodes there are on the output path, while the autoregressive models lack this information, implicitly tracking path length as they make successive token predictions. Second, both the masked completion objective and the iterative inference procedure allow the masked model to rely on local and bidirectional inference, but the autoregressive models are strictly past-to-future (i.e., start-to-goal). These additional supports that the masked models receive may give them the appearance of being stronger, but this appearance may be deceiving. In fact, recent work has noted that the autoregressive models may learn the underlying data generation structure more effectively than masked models (Allen-Zhu & Li, 2023).

In addition, we found that models that search over edge tokens likely require more complex architectures to perform effective in-context graph traversal. This result matches insights from recent work exploring the effectiveness of transformers on different graph reasoning tasks as well as grade-school math problems, suggesting that depth is a crucial factor for the capacity of multi-step reasoning (Sanford et al., 2024; Ye et al., 2024). Other work has also explored learning from more scaled-up versions of graph-traversal-like tasks and the consequences therein. For example, Vafa et al. (2024) trained transformers on trajectories from a New York taxi rides dataset, a kind of noisy shortest-path distribution in real life. Relatedly, Li et al. (2022) trained transformers to predict moves in board game trajectories (Othello). Both works showed that these models, although not explicitly instructed to learn any models of the environment (city maps or board game rules), seem to acquire an emergent world representation internally. But careful analysis using a variety of evaluation metrics shows that these representations may be incoherent and fragile (Vafa et al., 2024). In this context, it will be interesting to explore whether simple BC-based subgoal preferences transform to more explicit subgoal use when the model is tasked in a more agentic setting. This will allow us to understand how a learning system can leap from implicit heuristics to more explicitly leveraging the true structure of the underlying environment in active problem solving.

## CONCLUSION

We used neural networks to explore if learning to efficiently traverse graph-like environments leads models to acquire human-like subgoal preferences. When trained to predict shortest paths in these environments, models such as transformers and LSTMs develop traversal preferences consistent with human subgoal preferences found in prior work. The model decision process and learning dynamics provide new hypotheses about human subgoal discovery, suggesting that sophisticated planning abilities can originate from efficient computational processes and the approximation of optimal solutions that reflect the environment structure.

## Acknowledgments

We would like to thank members of the Stanford PDP lab for useful discussions that helped shape this work. We are also grateful for the feedback from anonymous reviewers for helping to strengthen this paper.

## Funding Information

The authors received no specific funding for this work.

## Authors Contributions

Y.L.: Conceptualization; Data curation; Formal analysis; Investigation; Methodology; Project administration; Software; Validation; Visualization; Writing – original draft; Writing – review & editing. J.L.M.: Conceptualization; Investigation; Methodology; Project administration; Resources; Supervision; Validation; Writing – original draft; Writing – review & editing.

## Data Availability Statement

The datasets used in this work can be found at: https://github.com/Effie-Li/graph-subgoal-public.
